# (1-{(*E*)-[Phen­yl(pyridin-2-yl-κ*N*)methyl­idene]amino-κ*N*}pyrrolidin-2-one-κ*O*)bis­(thio­cyanato-κ*N*)copper(II)

**DOI:** 10.1107/S1600536812039918

**Published:** 2012-09-26

**Authors:** Roji J. Kunnath, M. R. Prathapachandra Kurup, Seik Weng Ng

**Affiliations:** a Department of Applied Chemistry, Cochin University of Science and Technology, Kochi 682 022, India; b Department of Chemistry, University of Malaya, 50603 Kuala Lumpur, Malaysia; c Chemistry Department, King Abdulaziz University, PO Box 80203 Jeddah, Saudi Arabia

## Abstract

The Cu^II^ atom in the title compound, [Cu(NCS)_2_(C_16_H_15_N_3_O)], is bonded to the N atoms of two thio­cyanate ions, and is *N*,*N*′-chelated by the Schiff base ligand. The four N atoms surround the metal atom to form a distorted square; the square environment is distorted towards a square pyramid by a long Cu⋯O inter­action. In the crystal, two C atoms of the pyrrolidin-2-one ring are disordered over two positions in a 1:1 ratio.

## Related literature

For the copper dichloride adduct of the Schiff base, see: Kunnath *et al.* (2012 ▶).
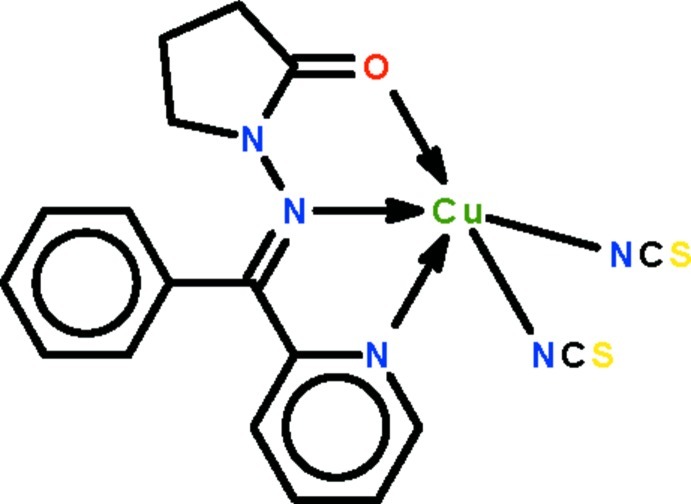


## Experimental

### 

#### Crystal data

[Cu(NCS)_2_(C_16_H_15_N_3_O)]
*M* *_r_* = 445.01
Monoclinic, 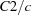

*a* = 11.8883 (3) Å
*b* = 13.3578 (3) Å
*c* = 23.9669 (6) Å
β = 98.596 (1)°
*V* = 3763.23 (16) Å^3^
*Z* = 8
Mo *K*α radiation
μ = 1.40 mm^−1^
*T* = 293 K
0.4 × 0.3 × 0.2 mm

#### Data collection

Bruker Kappa APEXII diffractometer
Absorption correction: multi-scan (*SADABS*; Sheldrick, 1996 ▶) *T*_min_ = 0.614, *T*_max_ = 1.000
7808 measured reflections
4232 independent reflections
3354 reflections with *I* > 2σ(*I*)
*R*_int_ = 0.033

#### Refinement

*R*[*F* ^2^ > 2σ(*F* ^2^)] = 0.064
*wR*(*F* ^2^) = 0.169
*S* = 1.16
4232 reflections
250 parameters
3 restraints
H-atom parameters constrained
Δρ_max_ = 0.73 e Å^−3^
Δρ_min_ = −0.63 e Å^−3^

### 

Data collection: *APEX2* (Bruker, 2010 ▶); cell refinement: *SAINT* (Bruker, 2010 ▶); data reduction: *SAINT*; program(s) used to solve structure: *SHELXS97* (Sheldrick, 2008 ▶); program(s) used to refine structure: *SHELXL97* (Sheldrick, 2008 ▶); molecular graphics: *X-SEED* (Barbour, 2001 ▶); software used to prepare material for publication: *publCIF* (Westrip, 2010 ▶).

## Supplementary Material

Crystal structure: contains datablock(s) global, I. DOI: 10.1107/S1600536812039918/xu5623sup1.cif

Structure factors: contains datablock(s) I. DOI: 10.1107/S1600536812039918/xu5623Isup2.hkl

Additional supplementary materials:  crystallographic information; 3D view; checkCIF report

## Figures and Tables

**Table 1:** Selected bond lengths (Å)

Cu1—O1	2.676 (4)
Cu1—N1	1.998 (4)
Cu1—N2	2.000 (3)
Cu1—N4	1.957 (4)
Cu1—N5	1.912 (4)

**Fig. 1.**
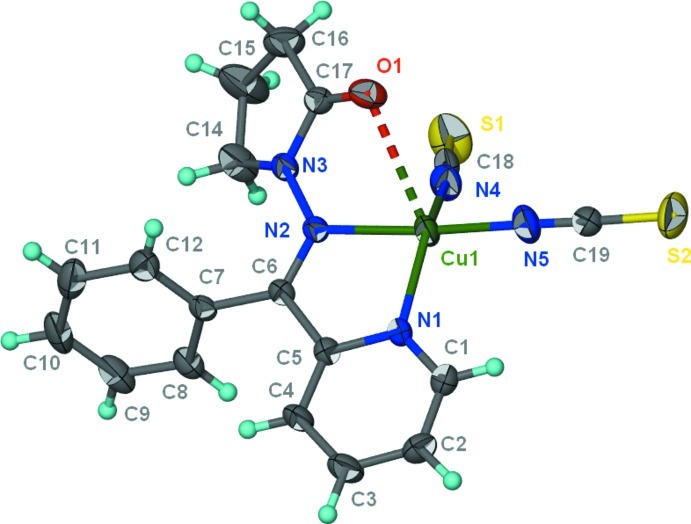
Thermal ellipsoid plot (Barbour, 2001) of Cu(NCS)2(C16H15N3O) at the 50% probability level; hydrogen atoms are drawn as spheres of arbitrary radius. The disorder in the pyrrolidine ring is not shown.

## supplementary crystallographic information

### Comment

1-[(*E*)-[Phenyl(pyridin-2-yl)methylidene]amino]pyrrolidine-2-one is a tridentate Schiff base that can only be synthesized *in situ*; it was isolated as its copper dichloride adduct in an earlier study (Kunnath *et al.*, 2012). In the present copper dithiocyanate adduct (Scheme I, Fig. 1), the geometry is also a square pyramid but the apical O atom lies at 2.676 (4) Å whereas the geometry of the copper dichloride analog is an almost undistorted square pyramid.

### Experimental

1-[(*E*)-[Phenyl(pyridin-2-yl)methylidene]amino]pyrrolidine-2-one was synthesized *in situ* from 2-benzoylpyridine (0.183 g, 1 mmol) and 1-aminopyrrolidin-2-one (0.100 g, 1 mmol) by heating the reactants in methanol for 2 hous. Copper(II) chloride dihydrate (0.170 g, 1 mmol) was added, and the mixture heated for 2 h. Sodium thiocyanate (0.194 g, 2 mmol) was added and the reaction was heated for another for 1 h. The resulting pale green solid was collected and recrystallized from alcohol.

### Refinement

Carbon-bound H-atoms were placed in calculated positions (C–H 0.93–0.97 Å) and were included in the refinement in the riding model approximation, with *U*(H) set to 1.2*U*(C).

Two of the methylene carbons in the pyrrolidine ring is disordered; the disorder was regarded as a 1:1 type of disorder. Pairs of C–C distances were restrained to within 0.01 Å of each other, and the temperature factors of the primed atoms were set to those of the unprimed ones.

### Crystal data

**Table:**

[Cu(NCS)_2_(C_16_H_15_N_3_O)]	*F*(000) = 1816
*M**_r_* = 445.01	*D*_x_ = 1.571 Mg m^−^^3^
Monoclinic, *C*2/*c*	Mo *K*α radiation, λ = 0.71073 Å
Hall symbol: -C 2yc	Cell parameters from 3798 reflections
*a* = 11.8883 (3) Å	θ = 2.3–28.2°
*b* = 13.3578 (3) Å	µ = 1.40 mm^−^^1^
*c* = 23.9669 (6) Å	*T* = 293 K
β = 98.596 (1)°	Prism, green
*V* = 3763.23 (16) Å^3^	0.4 × 0.3 × 0.2 mm
*Z* = 8	

### Data collection

**Table:**

Bruker Kappa APEXII diffractometer	4232 independent reflections
Radiation source: fine-focus sealed tube	3354 reflections with *I* > 2σ(*I*)
Graphite monochromator	*R*_int_ = 0.033
ω scans	θ_max_ = 27.5°, θ_min_ = 2.4°
Absorption correction: multi-scan (*SADABS*; Sheldrick, 1996)	*h* = −10→15
*T*_min_ = 0.614, *T*_max_ = 1.000	*k* = −17→15
7808 measured reflections	*l* = −28→31

### Refinement

**Table:**

Refinement on *F*^2^	Primary atom site location: structure-invariant direct methods
Least-squares matrix: full	Secondary atom site location: difference Fourier map
*R*[*F*^2^ > 2σ(*F*^2^)] = 0.064	Hydrogen site location: inferred from neighbouring sites
*wR*(*F*^2^) = 0.169	H-atom parameters constrained
*S* = 1.16	*w* = 1/[σ^2^(*F*_o_^2^) + (0.0468*P*)^2^ + 21.4376*P*] where *P* = (*F*_o_^2^ + 2*F*_c_^2^)/3
4232 reflections	(Δ/σ)_max_ = 0.001
250 parameters	Δρ_max_ = 0.73 e Å^−^^3^
3 restraints	Δρ_min_ = −0.63 e Å^−^^3^

### Fractional atomic coordinates and isotropic or equivalent isotropic displacement parameters (Å^2^)

**Table:**

	*x*	*y*	*z*	*U*_iso_*/*U*_eq_	Occ. (<1)
Cu1	0.38438 (5)	0.63120 (5)	0.47230 (2)	0.03878 (19)	
S1	0.08245 (15)	0.56173 (18)	0.32832 (8)	0.0762 (6)	
S2	0.20280 (18)	0.64256 (14)	0.63148 (7)	0.0733 (5)	
N1	0.4851 (3)	0.7508 (3)	0.48791 (15)	0.0313 (8)	
N2	0.4894 (3)	0.6106 (3)	0.41547 (14)	0.0301 (8)	
N3	0.4722 (3)	0.5290 (3)	0.38127 (15)	0.0324 (8)	
N4	0.2510 (3)	0.5904 (4)	0.41915 (19)	0.0467 (10)	
N5	0.3145 (4)	0.6345 (4)	0.53914 (19)	0.0524 (11)	
O1	0.4251 (3)	0.4376 (3)	0.45491 (14)	0.0466 (9)	
C1	0.4875 (4)	0.8152 (4)	0.5301 (2)	0.0389 (10)	
H1	0.4366	0.8067	0.5556	0.047*	
C2	0.5623 (5)	0.8943 (4)	0.5375 (2)	0.0458 (12)	
H2	0.5616	0.9381	0.5676	0.055*	
C3	0.6381 (5)	0.9080 (4)	0.5002 (2)	0.0497 (13)	
H3	0.6890	0.9613	0.5043	0.060*	
C4	0.6367 (4)	0.8401 (4)	0.4559 (2)	0.0423 (11)	
H4	0.6878	0.8467	0.4303	0.051*	
C5	0.5592 (4)	0.7635 (3)	0.45059 (17)	0.0311 (9)	
C6	0.5531 (3)	0.6846 (3)	0.40697 (17)	0.0293 (9)	
C7	0.6212 (4)	0.6942 (3)	0.35987 (18)	0.0315 (9)	
C8	0.6005 (4)	0.7732 (4)	0.3226 (2)	0.0407 (11)	
H8	0.5484	0.8226	0.3285	0.049*	
C9	0.6573 (5)	0.7787 (5)	0.2767 (2)	0.0518 (14)	
H9	0.6419	0.8309	0.2510	0.062*	
C10	0.7366 (5)	0.7074 (5)	0.2687 (2)	0.0563 (16)	
H10	0.7751	0.7118	0.2377	0.068*	
C11	0.7595 (5)	0.6294 (5)	0.3064 (2)	0.0563 (15)	
H11	0.8142	0.5820	0.3011	0.068*	
C12	0.7012 (4)	0.6214 (4)	0.3520 (2)	0.0424 (11)	
H12	0.7153	0.5682	0.3771	0.051*	
C14	0.4536 (5)	0.5269 (5)	0.3197 (2)	0.0565 (16)	
H14A	0.5225	0.5440	0.3047	0.068*	0.50
H14B	0.3934	0.5726	0.3044	0.068*	0.50
H14C	0.5252	0.5209	0.3051	0.068*	0.50
H14D	0.4150	0.5870	0.3044	0.068*	0.50
C15	0.420 (2)	0.4208 (13)	0.3069 (19)	0.064 (7)	0.50
H15A	0.4813	0.3862	0.2921	0.077*	0.50
H15B	0.3530	0.4187	0.2784	0.077*	0.50
C16	0.396 (5)	0.370 (3)	0.3602 (13)	0.051 (5)	0.50
H16A	0.3157	0.3546	0.3581	0.062*	0.50
H16B	0.4393	0.3082	0.3670	0.062*	0.50
C15'	0.381 (2)	0.4368 (14)	0.3052 (19)	0.064 (7)	0.50
H15C	0.4003	0.4035	0.2719	0.077*	0.50
H15D	0.3011	0.4539	0.2991	0.077*	0.50
C16'	0.411 (5)	0.372 (3)	0.3574 (13)	0.051 (5)	0.50
H16C	0.3491	0.3279	0.3623	0.062*	0.50
H16D	0.4787	0.3326	0.3549	0.062*	0.50
C17	0.4330 (4)	0.4452 (3)	0.40513 (19)	0.0343 (10)	
C18	0.1816 (4)	0.5783 (4)	0.3809 (2)	0.0427 (12)	
C19	0.2681 (4)	0.6390 (4)	0.5774 (2)	0.0394 (10)	

### Atomic displacement parameters (Å^2^)

**Table:**

	*U*^11^	*U*^22^	*U*^33^	*U*^12^	*U*^13^	*U*^23^
Cu1	0.0356 (3)	0.0437 (3)	0.0407 (3)	−0.0122 (3)	0.0179 (2)	−0.0071 (3)
S1	0.0496 (9)	0.1086 (16)	0.0666 (11)	−0.0120 (9)	−0.0039 (8)	0.0002 (10)
S2	0.0979 (13)	0.0745 (12)	0.0592 (9)	0.0200 (10)	0.0498 (9)	0.0077 (8)
N1	0.0292 (18)	0.0318 (19)	0.0349 (19)	−0.0018 (15)	0.0118 (15)	−0.0001 (15)
N2	0.0351 (19)	0.0295 (19)	0.0273 (17)	−0.0065 (15)	0.0098 (14)	−0.0033 (14)
N3	0.0328 (19)	0.034 (2)	0.0315 (18)	−0.0083 (16)	0.0083 (15)	−0.0046 (15)
N4	0.032 (2)	0.055 (3)	0.054 (3)	−0.0064 (19)	0.008 (2)	−0.006 (2)
N5	0.044 (2)	0.064 (3)	0.054 (3)	−0.015 (2)	0.024 (2)	−0.008 (2)
O1	0.065 (2)	0.0375 (19)	0.0373 (18)	−0.0111 (17)	0.0072 (16)	0.0039 (14)
C1	0.040 (2)	0.038 (3)	0.042 (3)	0.003 (2)	0.014 (2)	−0.003 (2)
C2	0.057 (3)	0.036 (3)	0.046 (3)	−0.002 (2)	0.012 (2)	−0.012 (2)
C3	0.060 (3)	0.042 (3)	0.050 (3)	−0.024 (3)	0.015 (2)	−0.007 (2)
C4	0.046 (3)	0.036 (3)	0.048 (3)	−0.018 (2)	0.018 (2)	−0.005 (2)
C5	0.032 (2)	0.031 (2)	0.032 (2)	−0.0017 (17)	0.0088 (17)	−0.0001 (17)
C6	0.028 (2)	0.028 (2)	0.032 (2)	−0.0042 (17)	0.0074 (17)	−0.0001 (17)
C7	0.032 (2)	0.031 (2)	0.033 (2)	−0.0118 (18)	0.0076 (17)	−0.0056 (17)
C8	0.040 (3)	0.044 (3)	0.040 (2)	−0.010 (2)	0.011 (2)	0.003 (2)
C9	0.050 (3)	0.068 (4)	0.037 (3)	−0.026 (3)	0.006 (2)	0.002 (2)
C10	0.050 (3)	0.082 (4)	0.042 (3)	−0.031 (3)	0.023 (2)	−0.018 (3)
C11	0.044 (3)	0.069 (4)	0.060 (3)	−0.008 (3)	0.021 (3)	−0.026 (3)
C12	0.037 (2)	0.042 (3)	0.051 (3)	−0.005 (2)	0.012 (2)	−0.006 (2)
C14	0.069 (4)	0.069 (4)	0.031 (3)	−0.030 (3)	0.006 (2)	−0.004 (2)
C15	0.079 (17)	0.073 (7)	0.043 (4)	−0.040 (10)	0.021 (14)	−0.021 (7)
C16	0.060 (12)	0.041 (3)	0.053 (4)	−0.020 (5)	0.009 (5)	−0.012 (3)
C15'	0.079 (17)	0.073 (7)	0.043 (4)	−0.040 (10)	0.021 (14)	−0.021 (7)
C16'	0.060 (12)	0.041 (3)	0.053 (4)	−0.020 (5)	0.009 (5)	−0.012 (3)
C17	0.030 (2)	0.031 (2)	0.042 (2)	−0.0041 (18)	0.0071 (18)	−0.0042 (19)
C18	0.035 (3)	0.042 (3)	0.056 (3)	0.000 (2)	0.023 (2)	0.002 (2)
C19	0.039 (2)	0.036 (2)	0.045 (3)	−0.002 (2)	0.012 (2)	−0.002 (2)

### Geometric parameters (Å, º)

**Table:**

Cu1—O1	2.676 (4)	C8—C9	1.376 (7)
Cu1—N1	1.998 (4)	C8—H8	0.9300
Cu1—N2	2.000 (3)	C9—C10	1.373 (9)
Cu1—N4	1.957 (4)	C9—H9	0.9300
Cu1—N5	1.912 (4)	C10—C11	1.380 (9)
S1—C18	1.606 (6)	C10—H10	0.9300
S2—C19	1.608 (5)	C11—C12	1.383 (7)
N1—C1	1.324 (6)	C11—H11	0.9300
N1—C5	1.357 (5)	C12—H12	0.9300
N2—C6	1.280 (5)	C14—C15	1.490 (11)
N2—N3	1.361 (5)	C14—C15'	1.490 (11)
N3—C17	1.370 (6)	C14—H14A	0.9700
N3—C14	1.460 (6)	C14—H14B	0.9700
N4—C18	1.150 (7)	C14—H14C	0.9700
N5—C19	1.141 (6)	C14—H14D	0.9700
O1—C17	1.215 (5)	C15—C16	1.516 (18)
C1—C2	1.376 (7)	C15—H15A	0.9700
C1—H1	0.9300	C15—H15B	0.9700
C2—C3	1.374 (7)	C16—C17	1.492 (9)
C2—H2	0.9300	C16—H16A	0.9700
C3—C4	1.395 (7)	C16—H16B	0.9700
C3—H3	0.9300	C15'—C16'	1.516 (18)
C4—C5	1.369 (6)	C15'—H15C	0.9700
C4—H4	0.9300	C15'—H15D	0.9700
C5—C6	1.478 (6)	C16'—C17	1.493 (9)
C6—C7	1.489 (6)	C16'—H16C	0.9700
C7—C12	1.393 (7)	C16'—H16D	0.9700
C7—C8	1.381 (7)		
			
N5—Cu1—N4	98.09 (19)	C11—C10—H10	119.8
N5—Cu1—N1	98.49 (17)	C12—C11—C10	120.2 (6)
N4—Cu1—N1	138.54 (18)	C12—C11—H11	119.9
N5—Cu1—N2	165.37 (19)	C10—C11—H11	119.9
N4—Cu1—N2	92.65 (16)	C7—C12—C11	119.1 (5)
N1—Cu1—N2	79.61 (14)	C7—C12—H12	120.5
N5—Cu1—O1	105.33 (17)	C11—C12—H12	120.5
N4—Cu1—O1	77.12 (16)	N3—C14—C15	102.9 (18)
N1—Cu1—O1	133.21 (13)	N3—C14—C15'	104.2 (18)
N2—Cu1—O1	67.36 (13)	N3—C14—H14A	111.2
C1—N1—C5	118.6 (4)	C15—C14—H14A	111.2
C1—N1—Cu1	127.3 (3)	N3—C14—H14B	111.2
C5—N1—Cu1	114.0 (3)	C15—C14—H14B	111.2
C6—N2—N3	124.2 (3)	H14A—C14—H14B	109.1
C6—N2—Cu1	116.5 (3)	N3—C14—H14C	110.9
N3—N2—Cu1	117.9 (3)	C15'—C14—H14C	110.9
N2—N3—C17	115.7 (3)	N3—C14—H14D	110.9
N2—N3—C14	127.7 (4)	C15'—C14—H14D	110.9
C17—N3—C14	113.4 (4)	H14C—C14—H14D	108.9
C18—N4—Cu1	166.7 (4)	C14—C15—C16	109 (3)
C19—N5—Cu1	176.4 (5)	C14—C15—H15A	109.8
C17—O1—Cu1	96.6 (3)	C16—C15—H15A	109.8
N1—C1—C2	122.6 (4)	C14—C15—H15B	109.8
N1—C1—H1	118.7	C16—C15—H15B	109.8
C2—C1—H1	118.7	H15A—C15—H15B	108.2
C3—C2—C1	119.5 (5)	C17—C16—C15	103 (3)
C3—C2—H2	120.3	C17—C16—H16A	111.1
C1—C2—H2	120.3	C15—C16—H16A	111.1
C2—C3—C4	118.3 (5)	C17—C16—H16B	111.1
C2—C3—H3	120.8	C15—C16—H16B	111.1
C4—C3—H3	120.8	H16A—C16—H16B	109.1
C5—C4—C3	119.2 (4)	C14—C15'—C16'	102 (3)
C5—C4—H4	120.4	C14—C15'—H15C	111.4
C3—C4—H4	120.4	C16'—C15'—H15C	111.4
N1—C5—C4	121.8 (4)	C14—C15'—H15D	111.4
N1—C5—C6	114.1 (4)	C16'—C15'—H15D	111.4
C4—C5—C6	124.0 (4)	H15C—C15'—H15D	109.2
N2—C6—C5	113.9 (4)	C17—C16'—C15'	105 (3)
N2—C6—C7	126.0 (4)	C17—C16'—H16C	110.8
C5—C6—C7	120.0 (4)	C15'—C16'—H16C	110.8
C12—C7—C8	120.3 (4)	C17—C16'—H16D	110.8
C12—C7—C6	120.1 (4)	C15'—C16'—H16D	110.8
C8—C7—C6	119.5 (4)	H16C—C16'—H16D	108.9
C9—C8—C7	119.8 (5)	O1—C17—N3	124.0 (4)
C9—C8—H8	120.1	O1—C17—C16	126.4 (19)
C7—C8—H8	120.1	N3—C17—C16	109.5 (18)
C10—C9—C8	120.3 (5)	O1—C17—C16'	131.9 (18)
C10—C9—H9	119.9	N3—C17—C16'	104.1 (18)
C8—C9—H9	119.9	N4—C18—S1	178.7 (5)
C9—C10—C11	120.3 (5)	N5—C19—S2	178.7 (5)
C9—C10—H10	119.8		
			
N5—Cu1—N1—C1	−6.5 (4)	N3—N2—C6—C7	−1.5 (7)
N4—Cu1—N1—C1	106.1 (4)	Cu1—N2—C6—C7	−167.7 (3)
N2—Cu1—N1—C1	−171.8 (4)	N1—C5—C6—N2	−8.3 (6)
O1—Cu1—N1—C1	−127.0 (4)	C4—C5—C6—N2	167.9 (5)
N5—Cu1—N1—C5	172.4 (3)	N1—C5—C6—C7	173.9 (4)
N4—Cu1—N1—C5	−75.0 (4)	C4—C5—C6—C7	−9.9 (7)
N2—Cu1—N1—C5	7.1 (3)	N2—C6—C7—C12	−57.0 (6)
O1—Cu1—N1—C5	51.9 (4)	C5—C6—C7—C12	120.5 (5)
N5—Cu1—N2—C6	−96.2 (8)	N2—C6—C7—C8	120.0 (5)
N4—Cu1—N2—C6	126.5 (3)	C5—C6—C7—C8	−62.5 (6)
N1—Cu1—N2—C6	−12.4 (3)	C12—C7—C8—C9	1.6 (7)
O1—Cu1—N2—C6	−158.6 (4)	C6—C7—C8—C9	−175.4 (4)
N5—Cu1—N2—N3	96.7 (7)	C7—C8—C9—C10	−1.8 (7)
N4—Cu1—N2—N3	−40.6 (3)	C8—C9—C10—C11	0.5 (8)
N1—Cu1—N2—N3	−179.5 (3)	C9—C10—C11—C12	1.1 (8)
O1—Cu1—N2—N3	34.3 (3)	C8—C7—C12—C11	−0.1 (7)
C6—N2—N3—C17	159.9 (4)	C6—C7—C12—C11	176.9 (4)
Cu1—N2—N3—C17	−34.1 (5)	C10—C11—C12—C7	−1.3 (8)
C6—N2—N3—C14	−42.1 (7)	N2—N3—C14—C15	−170.4 (12)
Cu1—N2—N3—C14	123.9 (5)	C17—N3—C14—C15	−12.0 (13)
N5—Cu1—N4—C18	144.6 (19)	N2—N3—C14—C15'	−150.3 (13)
N5—Cu1—O1—C17	159.6 (3)	C17—N3—C14—C15'	8.1 (13)
N4—Cu1—O1—C17	64.6 (3)	N3—C14—C15—C16	12 (3)
N1—Cu1—O1—C17	−82.6 (3)	C15'—C14—C15—C16	−84 (10)
N2—Cu1—O1—C17	−33.9 (3)	C14—C15—C16—C17	−8 (4)
C5—N1—C1—C2	−0.2 (7)	N3—C14—C15'—C16'	−25 (3)
Cu1—N1—C1—C2	178.7 (4)	C15—C14—C15'—C16'	63 (10)
N1—C1—C2—C3	0.0 (8)	C14—C15'—C16'—C17	34 (4)
C1—C2—C3—C4	−0.5 (9)	Cu1—O1—C17—N3	30.6 (5)
C2—C3—C4—C5	1.1 (9)	Cu1—O1—C17—C16	−145 (3)
C1—N1—C5—C4	0.9 (7)	Cu1—O1—C17—C16'	−153 (3)
Cu1—N1—C5—C4	−178.1 (4)	N2—N3—C17—O1	−8.0 (7)
C1—N1—C5—C6	177.1 (4)	C14—N3—C17—O1	−169.1 (5)
Cu1—N1—C5—C6	−1.9 (5)	N2—N3—C17—C16	169 (3)
C3—C4—C5—N1	−1.3 (8)	C14—N3—C17—C16	7 (3)
C3—C4—C5—C6	−177.2 (5)	N2—N3—C17—C16'	175 (3)
N3—N2—C6—C5	−179.2 (4)	C14—N3—C17—C16'	14 (3)
Cu1—N2—C6—C5	14.6 (5)	C15—C16—C17—O1	177.0 (18)
